# Skeletal muscle: molecular structure, myogenesis, biological functions, and diseases

**DOI:** 10.1002/mco2.649

**Published:** 2024-07-10

**Authors:** Lan‐Ting Feng, Zhi‐Nan Chen, Huijie Bian

**Affiliations:** ^1^ Department of Cell Biology & National Translational Science Center for Molecular Medicine National Key Laboratory of New Drug Discovery and Development for Major Diseases Fourth Military Medical University Xi'an China

**Keywords:** biological function, myogenesis, myonuclear, myopathy, skeletal muscle

## Abstract

Skeletal muscle is an important motor organ with multinucleated myofibers as its smallest cellular units. Myofibers are formed after undergoing cell differentiation, cell–cell fusion, myonuclei migration, and myofibril crosslinking among other processes and undergo morphological and functional changes or lesions after being stimulated by internal or external factors. The above processes are collectively referred to as myogenesis. After myofibers mature, the function and behavior of skeletal muscle are closely related to the voluntary movement of the body. In this review, we systematically and comprehensively discuss the physiological and pathological processes associated with skeletal muscles from five perspectives: molecule basis, myogenesis, biological function, adaptive changes, and myopathy. In the molecular structure and myogenesis sections, we gave a brief overview, focusing on skeletal muscle‐specific fusogens and nuclei‐related behaviors including cell–cell fusion and myonuclei localization. Subsequently, we discussed the three biological functions of skeletal muscle (muscle contraction, thermogenesis, and myokines secretion) and its response to stimulation (atrophy, hypertrophy, and regeneration), and finally settled on myopathy. In general, the integration of these contents provides a holistic perspective, which helps to further elucidate the structure, characteristics, and functions of skeletal muscle.

## Graphical abstract

The article systematically and comprehensively reviews the physiological and pathological processes associated with skeletal muscles from five perspectives: molecule basis, myogenesis, biological function, poststimulation response, and myopathy. We primarily focus on nuclei‐related behaviors of skeletal muscle, cell–cell fusion, and nuclei migration in the first two sections and discuss the three biological functions of skeletal muscle (muscle contraction, thermogenesis, and myokines secretion) and its response to stimulation (atrophy, hypertrophy, and regeneration), and finally settle on myopathy.

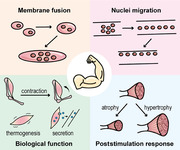

## 1 INTRODUCTION

Skeletal muscle is one of the three major muscle tissues of the human body. It is attached to the bones through the tendon and dominates all voluntary movements. From an evolutionary perspective, the morphology and function of skeletal muscles were confirmed 500−600 million years ago and have been preserved and developed throughout evolution.^1^ Researchers have observed muscle structure in the 560 million years old Haootia quadriformis fossil found in Newfoundland, Canada, which contributes to tracing the earliest origin of muscle tissue in animals.^2^ The conservation of evolution has led to extensive research on skeletal muscles in various experimental animals.

As one of the few physiological syncytia that can survive for a long time, myofiber has a unique system to support its generation and function. Generalized myogenesis covers the entire process from cell differentiation in the embryonic period, myofiber maturation to muscle morphology, and functional changes or lesions affected by internal or external factors in the adult phase.^3^, ^4^ Among them, cell–cell fusion mediated by skeletal muscle‐specific fusogens and myonuclei localization are the basis for ensuring the formation and stable operation of multinucleated myofiber.^5^, ^6^ Mature skeletal muscles play an important role in movement, respiration, body temperature maintenance, and organ protection, and their biological functions are mainly manifested in muscle contraction, thermogenesis, and secretion of myokines.^7^ In addition, under normal circumstances, the state of skeletal muscle tissue remains stable. However, muscle tissue undergoes atrophy, hypertrophy, or even myofiber death owing to exercise, injury, disease, or aging.^8^, ^9^, ^10^ Many of these processes include imbalances in the synthesis and degradation of substances as well as changes in the number of nuclei.^8^, ^11^ When the above processes are abnormal or unbalanced, the function of skeletal muscle is impaired and cause diseases, thus affecting the body's activity and causing respiratory depression or even death.

**FIGURE 1:**
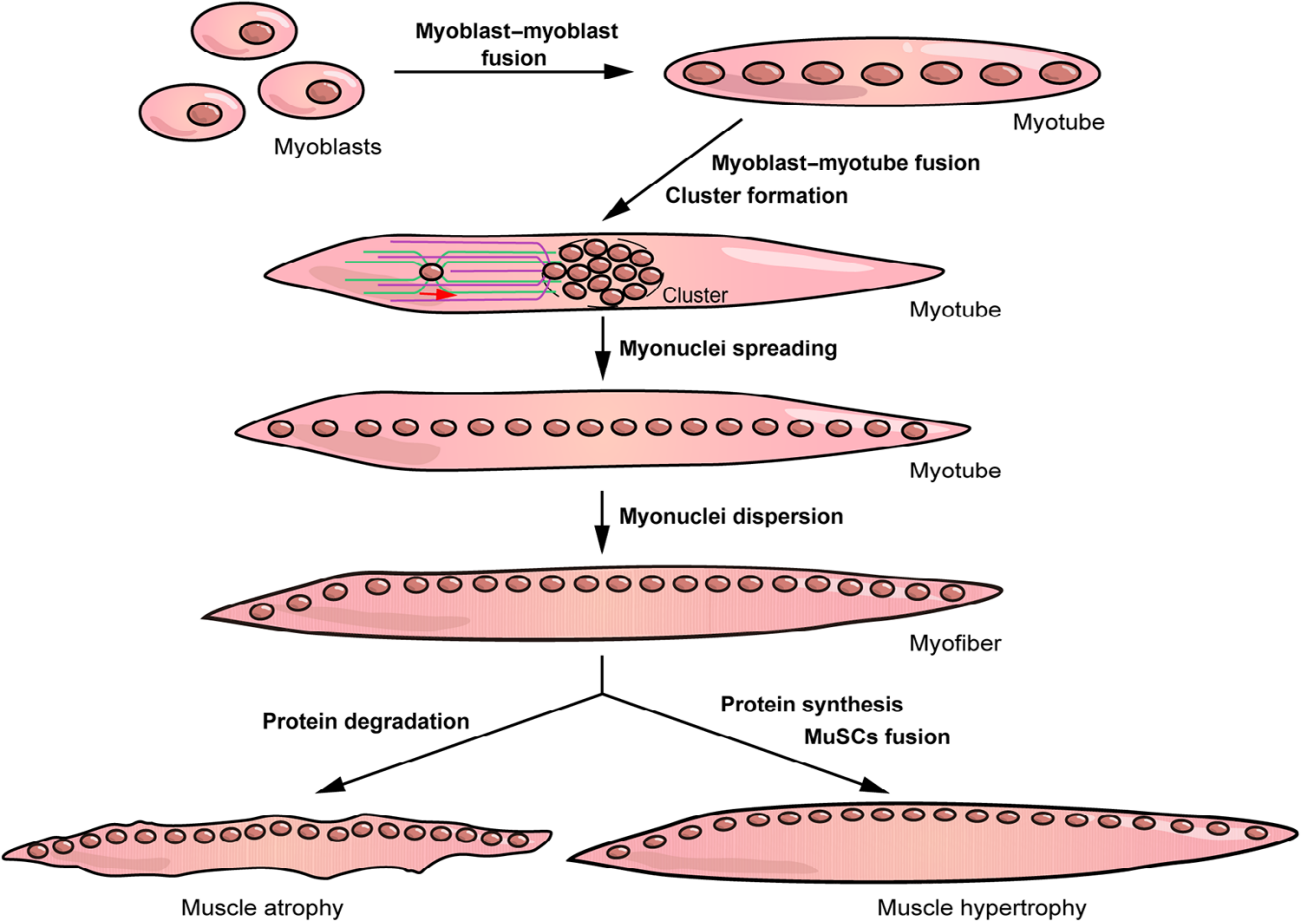
The formation process and state change of myofibers. During the embryonic period, myoblasts differentiate from precursor cells and fuse to form nascent myotubes. Subsequently, the myoblasts continue to fuse with the myotubes to expand and increase the number of myonuclei. Subsequently, nuclear aggregates are newly added to the center under the traction of the cytoskeleton and form a cluster. Then, myonuclei become spatially distant from one another and are eventually transferred to the plasma membrane. The migrated myonuclei are fixed and uniformly distributed at appropriate distances to maximize their function. The skeletal muscle state changes under stimulation. At the cellular level, this primarily depends on the relationship between protein synthesis and degradation. When protein synthesis is less than degradation, the skeletal muscle shows an atrophied state; otherwise, it shows hypertrophy. Additionally, muscle hypertrophy is associated with MuSC fusion. Muscle regeneration mediated by MuSCs that localize between myofibers can partially or even completely restore atrophied and damaged muscle tissue, which is crucial for recovery from injury and disease (not shown in the figure).

In this review, we systematically describe the physiological and pathological processes associated with skeletal muscle. In the first two sections, we cover the molecular basis and basic processes of myogenesis and primarily focus on nuclei‐related behaviors including cell–cell fusion and myonuclei localization (Figure 1). Subsequently, we discuss the biological function and poststimulation response of skeletal muscles. The former is performed via three processes: contraction, metabolism, and endocrine, whereas the latter constitutes muscle atrophy, hypertrophy, and regeneration (Figure 1). Finally, we discuss several myopathies related to cell fusion, myonuclei localization, and muscle atrophy. This review systematically and comprehensively identifies the relevant contents of skeletal muscles, which elucidates the structure, characteristics, and functions of skeletal muscles, and provides alternative targets for the treatment of myopathies.

## 2 MOLECULAR STRUCTURE OF THE SKELETAL MUSCLE SYSTEM

The skeletal muscle is a remarkable component of the motion system, playing a vital role in the rapid directional movement of animals. The smallest cell unit that constitutes skeletal muscle tissue is the myocyte, also known as myofiber. Numerous myofibers are closely arranged in bundles, complementary in length, and form muscle bundles by wrapping the perimysium on the surface. Multiple muscle bundles gather to form the complete skeletal muscle. Myofibers are syncytia in the physiological state, and their behavior is supported by various muscle‐specific molecules. In terms of myofiber formation, cell–cell fusion is an essential process that distinguishes myofibers from other single‐nuclear cells in the body and is mediated by two muscle‐specific and evolutionarily conserved proteins: myomaker (TMEM8c)^12^ and myomerger/myomixer/minion.^13^, ^14^, ^15^ In mature myofibers, myofibrils are an important component and their basic unit is sarcomere. The sarcomere is composed of four different myofilament systems: thick filament, thin filament, titin, and nebulin, which cooperate with each other to support the contractile function of skeletal muscle.^16^ To maintain the coherence and fluency of the content, myofilament systems and other molecules that play a role in inducing differentiation or regulating expression in myogenesis will be introduced in the corresponding sections. Therefore, in this section, we mainly focus on the structure and regulation of skeletal muscle‐specific fusogens: myomaker and myomerger.

### 2.1 Structure of skeletal muscle‐specific fusogens

**FIGURE 2:**
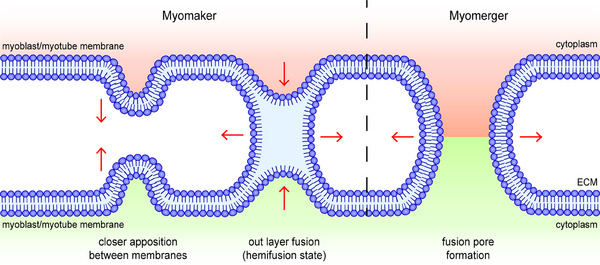
Putative membrane fusion process mediated by myomakers and myomergers. After the fusogen is anchored on another membrane involved in fusion (not shown in the figure), the anchored membranes overcome the hydrophilic resistance and continue to approach each other with the allosteric action of the fusogen. Subsequently, outer layers of anchored membranes fuse to form the hemifusion state, promoting the convergence of inner membrane layers and facilitating their fusion. A fusion pore is formed and continuously expands, and irreversible membrane fusion is completed after cytoplasmic exchange. In myoblast–myoblast and myoblast–myotube fusion, myomakers mediate the processes preceding fusion pore formation, whereas myomergers induce the remaining steps.

The microscopic process of membrane fusion can be roughly divided into six steps: (1) fusogen‐induced recognition and adhesion of target cells, (2) the formation of fusogetic complex anchored on both sides of target membranes, (3) the close apposition between the anchored membranes caused by the deformation of the fusogetic complex, (4) fusion between the out layers of anchored membranes (hemifusion state), (5) the formation and expansion of fusion pore, and (6) irreversible membrane fusion and cytoplasmic exchange^17^ (Figure 2). Therefore, fusogens are the most essential factors and identity markers in different types of syncytia formation and are the most direct and effective targets for human intervention in the fusion process.

#### 2.1.1 Myomaker

**FIGURE 3:**
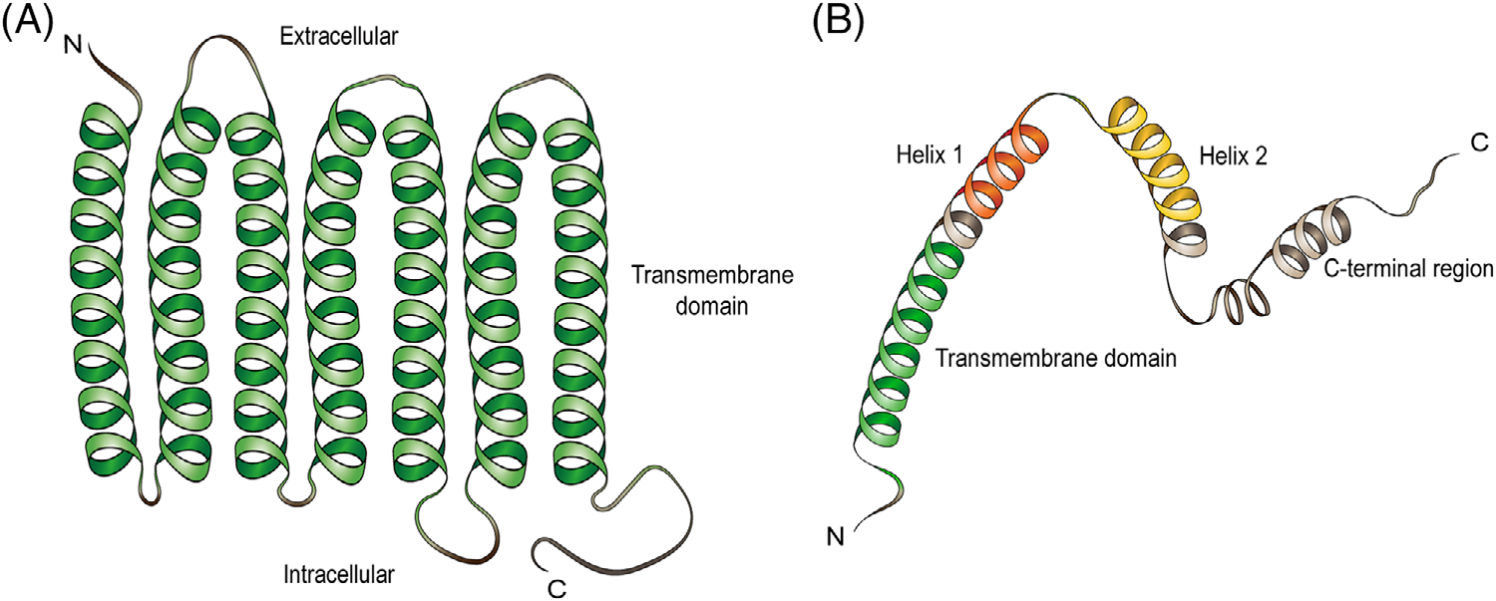
Structure of myomakers and myomergers. (A) Myomakers contain seven transmembrane domains and a 25‐amino acid intracellular C‐terminal tail, as indicated by myomaker topology (modified from Ref. 23). (B) Myomergers have only one transmembrane domain, an N‐terminal hydrophobic domain, and a C‐terminal domain containing helix‐1 and helix‐2, with a total length of 84 amino acids (modified from Ref. 31).

Myomaker, a muscle‐specific plasma membrane protein, is a limiting factor for myofiber fusion, and its molecular structure is highly conserved in vertebrates such as mice, chickens, zebrafish, trout, gilthead sea bream, and Japanese flounder.^12^, ^18^, ^19^, ^20^, ^21^, ^22^ According to the model of myomaker topology, myomakers contain seven transmembrane domains and a 25‐amino acid intracellular C‐terminal tail with a C‐A‐A‐X motif, which is necessary for the fusion process, possibly because the three cysteine residues at the C‐terminal domain function as lipidation sites^12^, ^23^ (Figure 3A). In cultured C2C12 myoblast cells in vitro, myomakers exist not only on the plasma membrane but also in Glogi and post‐Glogi vesicles, and C‐terminal palmitoylation of myomakers is important for their localization on the Glogi.^24^ However, the mechanism underlying myomaker transfer from Glogi and post‐Glogi vesicles to the cell membrane remains unknown. The timing of myomaker expression is under strict regulation (details provided below). Researchers have found excessive fusion of myoblasts and the formation of myofibers with an abnormally large number of nuclei in zebrafish embryos overexpressing myomaker.^20^ In contrast to myogenesis, the fusion process is completely dependent on the myomaker expressed on differentiated MuSCs during muscle hypertrophy and regeneration, whereas the myomaker expressed on myofibers has little effect on fusion dynamics.^11^, ^25^ Therefore, appropriate timing and location of myomaker expression are important for normal skeletal muscle formation.

#### 2.1.2 Myomerger

The function of myomergers in myofiber fusion was initially discovered in mice by three independent teams and then verified in many fish animals.^13^, ^14^, ^15^, ^26^, ^27^, ^28^, ^29^ Myomerger is a single‐pass transmembrane microprotein with a total length of 84 amino acids, encoded by the small open reading frame (smORF) Gm7325^13^, ^15^ (Figure 3B). Interestingly, the myomerger gene encodes two isoforms: a short isoform of 84 amino acids encoded by a shorter transcript with a single exon and a long isoform of 108 amino acids encoded by a longer transcript with an upstream exon replacing the transcriptional start site.^14^, ^30^ The former is the mainly expressed isoform during myoblast differentiation, and several essential domains have been identified, including an N‐terminal hydrophobic domain, which is believed to be the membrane anchoring region and a C‐terminal domain containing two α‐helices (helix‐1 and helix‐2)^13^ (Figure 3B). Amphipathic helix‐1 is located upstream of helix‐2 and is disordered when the myomerger is inactivated, whereas the hydrophobic helix‐2 can be inserted into the acyl tail region of the cell membrane.^31^ Previous experiments have shown that C‐terminal helices are dispensable for fusion and seem to be a legacy of evolution; however, the hydrophobic AxLyCxL motif in helix‐2 is considered to be a fusion peptide.^13^, ^30^ Nevertheless, recent research has indicated that helix‐1 and helix‐2 in the C‐terminal region are the main forces for fusion pore formation, as described in detail below.^31^ The long isoform contains an additional 24‐amino acid extension at the N‐terminus, but deletion of this extra region does not seem to affect the fusion faculty of the myomerger.^30^

### 2.2 Regulation of myomaker and myomerger expression

Under physiological conditions, accurate regulation of myomaker and myomerger expression is important for preventing fusion disorders and ensuring normal myofiber formation. The expression of myomakers and myomergers on atrophic myofibers can destroy membrane integrity, cause membrane damage, and even aggravate the process of muscle atrophy.^25^, ^32^ Hence, when myomakers and myomergers detected, it is crucial to understand the types of cells in which they are expressed and the specific cellular locations within those cells where their expression occurs.

The transcription factors that regulate the expression of myomakers and myomergers are primarily myogenic regulatory factors (MRFs), including myoblast determination (MyoD), myogenin (MyoG), and myogenic regulatory factor 5 (Myf5). The E‐box elements located upstream of the myomaker transcription start site are the binding sites of the transcription factors MyoD and MyoG, which activate myomaker expression,^21^, ^33^, ^34^ and HEYL, which inhibits myomaker transcription.^35^ miR‐491 and miR‐140‐3p, serving as inhibitors, could bind to the 3′‐UTR of mouse myomaker mRNA to inhibit myomaker expression, whereas miR‐205 plays the same role in porcine myoblast fusion.^21^, ^36^, ^37^ Similar to myomakers, myomergers can also be upregulated by MyoD and MyoG and downregulated by miRNAs. In a study on yellowfin seabream, researchers found that MyoD1 and MyoD2 have different binding sites on the E‐box of the myomerger gene, with MyoD1 binding on M2 and MyoD2 binding on M5, which may suggest a different regulatory ability between the two.^38^ In addition, miR‐7 binds to the 3′‐UTR of human myomerger mRNA and inhibits its translation, which can be lowered by lncRNA OIP5‐AS1 to ensure the normal fusion function of myofibers.^39^ In contrast to mouse MyoD‐deficient myoblasts that retain a certain fusion ability, human MyoD‐deficient myoblasts show a collective absence of myomaker, myomerger, and MyoG, with a complete lack of fusion.^40^, ^41^ Notably, MyoD itself is sufficient to activate the expression of myomakers and myomergers at the beginning of the fusion process without relying on other members of the MRFs family.^41^

As a downstream target gene of MyoD, MyoG plays a role similar to that of MyoD at different stages of fusion; however, MyoG affects the expression of myomakers but not myomergers.^41^ Studies have also shown that myoblast fusion cannot occur in the absence of MyoG and that MyoG can upregulate the expression of MyoD1 in a feedback manner.^42^ The mechanism model of MyoD and MyoG has been proposed in both chicken and human myoblasts: MyoD initiates the early fusion by upregulating the expression of myomakers and myomergers; after that, the expression of MyoG increases and replaces MyoD after MyoD expression decreases at the late stage of myofiber formation, promoting the expression of myomakers to enhance the fusion process and enlarge muscle fiber size.^21^, ^41^ Notably, some members of the MRFs family, such as MyoD and Myf5, have both complementary and redundant mechanisms of action. When one of the crucial molecules (MyoD) is missed for abnormal reasons, other members that complement it (Myf5) fill the functional gaps and guarantee normal fusion function to a certain extent.^40^, ^41^

Both myomakers and myomergers require dynamic translocation on the plasma membrane before fusion to exert their fusogenic activity at the cell surface.^23^, ^43^ Casein kinase 2 (CK2) mediates this process of myomerger and catalyzes the hierarchical regulation of the functional complex composing of myomakers and myomergers, whose CK2α subunit is regarded as a molecular regulator of fusogenic proteins.^43^ In addition, myomakers and myomergers are highly expressed in myofilament pseudopods and are mainly dominated by the unconventional myosin Myo10, suggesting that the fusogenic activity of myomakers may depend on the muscle filopodia composed of Myo10.^44^ Interestingly, tunnel nanotubes formed by Myo10 are needed during osteoclast differentiation and are required for both fusing cells, which is similar to the role of myomakers in myoblast differentiation.^45^

Several other molecules have been shown to affect the expression of myomakers and myomergers. Interleukin‐4 (IL‐4) acts as a myoblast recruitment factor after myotube formation, binds to IL‐4Rα, and enhances the expression of MyoD, MyoG, and myomergers without affecting myomakers, which possess feedback regulation with NFATc2.^46^, ^47^ Because IL‐4 is mainly produced by Th2 cells, we can reasonably speculate that IL‐4 can serve as a stimulus fusion factor after muscle injury. Inhibition of the Notch pathway not only promotes myoblast differentiation, but also upregulates the expression of myomakers by making MyoG dominant in competition with HEYL.^35^ Melatonin produced in the pineal and extra‐pineal sites in mammals can downregulate the expression of myomakers and myomergers and inhibit the formation of fusion pores in vitro.^48^ Human antigen R (HuR), an RNA binding protein, promotes the differentiation of goat MuSCs through enhancing the stability of myomakers.^49^ Furthermore, the presence and content of Piezo1, NADPH oxidase 4 (Nox4), ten‐eleven translocation‐2 (Tet‐2), vitamin D, and drugs such as andrographolide are all associated with the expression of myomakers and/or myomergers, which affect myofiber fusion^50^, ^51^, ^52^, ^53^, ^54^, ^55^ (Table 1).

**TABLE 1:** Molecules that regulate the expression of myomaker and myomerger.

Molecules	Gene family	Function	References
Myomaker (TMEM8c)	Muscle‐specific fusogen	Mediate the formation of hemifusion state	^12^
Myomerger/myomixer/ minion	Muscle‐specific fusogen	Drive the formation and expansion of fusion pore	^13^, ^14^, ^15^
MyoD	MRF family	Bind to E‐box 1 element of myomaker and myomerger gene to upregulate their expression in the early stage of myofiber formation	^21^, ^33^
MyoG	MRF family	Bind to E‐box 1 element of myomaker and myomerger gene to upregulate their expression in the late stage of myofiber formation	^21^, ^33^, ^34^
HEYL	Transcriptional factor	Bind to E‐box element of myomaker gene to downregulate its expression	^35^
miR‐491	miR‐491	Bind to 3′‐UTR of mouse myomaker mRNA to inhibit myomaker expression	^36^
miR‐140‐3p	miR‐140	Bind to 3′‐UTR of mouse myomaker mRNA to inhibit myomaker expression	^21^
miR‐205	miR‐205	Bind to 3′‐UTR of porcine myomaker mRNA to inhibit myomaker expression	^37^
miR‐7	miR‐7	Bind to 3′‐UTR of human myomerger mRNA and inhibit myomerger translation. miR‐7 can be inhibited by LncRNA OIP5‐AS1	^39^
Myf5	MRF family	Complementary and redundant molecular of MyoD in regulation myomaker and myomerger expression	^40^
CK2	Protein kinase	Mediate the dynamic translocation of myomerger before fusion	^43^
Myo10	Unconventional myosin	Essential component of myofilament pseudopods where myomaker is highly expressed	^44^
IL‐4	Cytokine	Function as myoblast recruitment factor and enhance the expression of MyoD, MyoG, and Myomerger	^46^
Notch pathway	Signal pathway	Upregulate the expression of myomaker through making MyoG dominant in competition with HEYL	^35^
Melatonin	Hormone	Downregulate the expression of myomaker and myomerger	^48^
HuR	RNA binding protein	Enhance the stability of myomaker	^49^
Piezo1	Mechanosensitive ion channel	Maintain appropriate calcium influx during muscle contraction and contribute to myomaker expression in myotube	^50^
Nox4	NADPH oxidase	Nox4‐derived ROS is involved in myomaker expression and enhance myocyte fusion without affecting myoblast differentiation	^51^
Tet2	Ten‐eleven translocation methylcytosine dioxygenase	Contribute to demethylation of myomaker and MyoG promoters	^53^
Vitamin D	Vitamin	High levels of vitamin D decreases the expression of myomaker and myomerger	^55^
Andrographolide	Drug	Active epigenetic modulation through promoting histone methylation such as H3K4Me2, H3K4Me3, H3K36Me2	^52^
PS	Phosphatide	Interact with Bai1 and Stab2 receptor to provide “fuse me” signal and promote changes in myomerger structure and fusion pore formation	^31^, ^56^, ^57^, ^58^

Abbreviations: CK2, Casein kinase 2; HuR, human antigen R; Myf5, myogenic regulatory factor 5;MyoD, myoblast determination; MyoG, myogenin; Nox4, NADPH oxidase 4; PS, phosphatidylserine; Tet‐2, ten‐eleven translocation‐2.

## 3 MYOGENESIS

Skeletal muscle myogenesis is a long and complex process in amniotes. Broadly, it refers to how muscle is formed by muscle progenitor cells and myoblasts, occurring under four distinct phases, namely the embryonic (primary myogenesis), fetal (secondary myogenesis), neonatal, and adult phases.^3^ Because the differentiation‐related processes in myogenesis have been adequately reviewed, in this section, we will systematically introduce the processes of myogenesis and then focus on the nuclear‐related behaviors: cell–cell fusion and myonuclei localization.

### 3.1 Overview of myogenesis

Myogenesis during embryonic and fetal phases is involved in the process of muscle development. During primary myogenesis, skeletal muscle progenitor myoblasts are formed after the differentiation of epiblast, mesoderm progenitor, presomitic progenitor, and aPSM‐dermomyotomal progenitor cells. The sequential expression of Pax3/7 and Myf5 is a hallmark of embryonic myogenic initiation.^4^ Pax3^+^ dermomyotomal progenitors fuse with each other to form primary myofibers, laying the foundation for subsequent muscle formation.^3^ Upon entering the secondary myogenesis phase, a subset of Pax3^+^ dermomyotomal progenitors downregulate Pax3 and express Pax7, inducing the formation of fetal myofibers via mutual fusion or fusion with primary myofibers.^4^ The assembly of basal lamina and formation of myotendinous junction as well as neuromuscular junction also occur during the fetal period.^3^ The main components of basal lamina such as laminin and fibronectin can directly promote myofiber formation by enhancing myoblasts proliferation and adhesion.

After birth, myofibers specialize to form slow‐twitch (type I) fibers and fast‐twitch (type II, including IIa, IIb, and IIx subtypes) fibers, and a muscle satellite cell (MuSC) niche is established in the neonatal period.^4^ A subset of Pax7^+^ progenitors differentiates into MuSCs to maintain the regenerative capability of mature muscle tissue.^59^ MuSCs are stem cells in skeletal muscle tissues that have the potential for proliferation and differentiation. Previous studies have shown that Pax7 is only expressed in proliferating myoblasts and that its expression is rapidly downregulated during differentiation.^60^ Cells derived from the asymmetric proliferation of Pax7^+^ MuSCs can be divided into Pax7^+^/Myf5^+^ MuSCs and Pax7^+^/Myf5^−^ MuSCs (approximately 10% of the total).^61^ Pax7^+^/Myf5^+^ MuSCs are distributed on the apical surface and adhere to myofibers via M‐cadherin, whereas Pax7^+^/Myf5^−^ MuSCs are connected to the basal lamina surrounding myofibers through laminin receptor integrin α7β1.^61^ MuSC‐mediated cell–cell fusion is the only way to add nuclei to mature myofibers; therefore, the presence of MuSCs is essential for the maintenance of muscle status. Damage to Pax7^+^ MuSCs during prepubertal development results in lifelong defects in muscle tissue.^62^

In the adult phase, the internal environment of skeletal muscle tissue remains relatively stable, and skeletal muscle performs its biological functions, including controlling body movement, maintaining body posture, supporting the entry and exit points, protecting bones as well as organs, and participating in temperature regulation under normal circumstances. However, the homeostasis of skeletal muscle tissue will be broken under physiological or pathological stimulation, manifested as atrophy or hypertrophy, accompanied by myofiber regeneration and skeletal muscle repair. Many of these processes are related to the imbalance in the synthesis and degradation of substances as well as changes in the number of nuclei. The related processes will also be detailed in the following sections.

In summary, generalized myogenesis occurs throughout life. The state and behaviors of skeletal muscle in each period are closely related to the health of the body, and any error in any link may lead to the occurrence of certain related diseases.

### 3.2 Cell–cell fusion

From a holistic perspective, myofiber formation is a two‐phase process. After myoblasts differentiate from embryonic mesoderm cells during embryonic period or MuSCs after birth, the primary fusion stage occurs between myoblasts to form multinucleated cells, which are subsequently transformed into nascent myotubes. In the secondary fusion stage, individual myoblasts fuse with nascent myotubes to promote myotube growth. In simple terms, the formation of single myofibers includes the myoblast–myoblast and myoblast–myotube fusion stages (Figure 1). Myoblasts undergo migration, elongation, adhesion, and recognition processes before membrane fusion induced by fusogens, which have been well reviewed.^63^, ^64^ In this section, we mainly focus on the cell fusion process mediated by skeletal muscle‐specific fusogens as well as its influencing factors during myogenesis.

Synergistic activity between myomakers and myomergers is sufficient for efficient heterologous cell–cell fusion. Relevant studies have shown that mouse fibroblasts, cortical bone stem cells, and mesenchymal stromal cells transfected with myomakers can efficiently fuse with C2C12 myotubes in vitro.^18^, ^64^, ^65^ Fibroblast–fibroblast fusion can be observed in myomaker and myomerger coexpressing fibroblast cultures.^13^ However, the fusion mediated by myomakers and myomergers is asymmetric, as both cells need to express myomakers whereas only one cell needs to express myomergers.^14^, ^15^

At present, there are two main models, an interactive model and an independent model, that describe how myomakers and myomergers jointly mediate myoblast fusion. In the interactive model, myomergers associate with myomakers to enhance their activity. Coimmunoprecipitation experiments indicate that myomakers physically interact with myomergers.^13^, ^30^ However, the binding site between myomakers and myomergers remains unclear. Therefore, the independent model appears to be more convincing.

In the exploration of the precise fusion stage mediated by myomakers and myomergers, it was found that rather than forming a fusogenic complex, myomakers and myomergers independently undertake different processes during membrane fusion (Figure 2). Myomakers mainly play a role in the initiation of fusion and the formation of a hemifusion state, whereas myomergers drive the formation and expansion of fusion pores.^5^ The absence of a myomerger did not affect the formation of the hemifusion state mediated by the myomaker.^5^ In this process, the myomaker functions as a transitional fusogen alone, whereas the myomerger facilitates what was once thought to be a process of spontaneous completion. However, myomakers may not be able to anchor their extracellular domains to the membrane to fuse like traditional fusogens because of their short N‐terminus. The anchoring domain and allosteric process of myomakers in mediating membrane fusion still need further exploration, which may be through the analysis of their crystal structures. At present, the structures of mice and Ciona myomakers have been resolved (according to the Protein Data Bank) to facilitate further research. Through the establishment and analysis of a physical model, researchers have found that when the membrane concentration of the myomerger is maintained at a low level, the myomerger‐induced transition from hemifusion to the fusion pore is promoted and accomplished by shifting the spontaneous curvature of the outer layers of the plasma membrane to a more positive value.^66^ However, the transition is inhibited if the membrane concentration of the myomerger is increased considerably, and the mechanism controlling its membrane concentration within an appropriate range remains unknown.^66^ Recently, in membrane tension analysis of the fusion process, researchers have found that the membrane distribution level of myomergers is strongly related to membrane tension in the early stages of differentiation, which challenges the theory that myomergers only function after the formation of a hemifusion state.^67^

At present, research on the role of myomakers and myomergers in the membrane fusion process has mainly focused on the myoblast–myoblast fusion stage, with few studies on the myoblast–myotube fusion stage. Based on the same two fusogens, we speculate that the myoblast–myotube fusion stage also follows the independent model, with differences in detail, because myotubes have a larger cell surface area, and the number and distribution of myomakers and myomergers on the myotube are unknown. However, these differences require further investigation.

### 3.3 Migration, localization, and functional characteristics of myonuclei

As the fusion process proceeds, the volume of the myotubes gradually increases. Early formed myotubes have the characteristics of “centrally located nuclei and peripheral masses of forming contractile myofilaments.”^6^ However, the myonuclei are regularly arranged under the sarcolemma in myofibers. Therefore, the migration of myonuclei from the center of the cell to the lower part of the sarcolemma is essential for the transition from myotubes to myofibers. The number of nuclei that work in an orderly manner after myofiber formation is an interesting question. In this section, we describe the process of myonucleus migration in detail and briefly discuss their modes of function.

#### 3.3.1 Myonuclei migration and localization

In mature myofibers, nuclei are fixed and uniformly distributed at an appropriate distance to maximize the distance between myonuclei, reduce mutual inhibition, and minimize the transport distance to improve efficiency.^68^ The correct localization of myonuclei is of great significance for the normal function of myofibers, and its disorder is associated with various myopathies, such as Emery–Dreifuss muscular dystrophy (EDMD) and centronuclear myopathy.^69^ Therefore, the migration and localization of myonuclei are essential for the transformation of myotubes into myofibers.

From the late stage of myoblast–myotube fusion, myonuclei must undergo cluster formation, myonuclear spreading, and myonuclear dispersion to reach their functional position^70^, ^71^ (Figure 1). These steps are mainly induced and regulated by microtubules (MT), MT motor proteins (including kinesin and dynein), MT‐associated proteins (MAPs), actin, actin‐associated proteins, and linkers of the nucleoskeleton and cytoskeleton (LINC) complex. Among them, MT and actin serve as the track for myonuclei operation, and MT motor proteins bind to the KASH domain of the LINC complex and transport the nuclei along the track in different directions.

**FIGURE 4:**
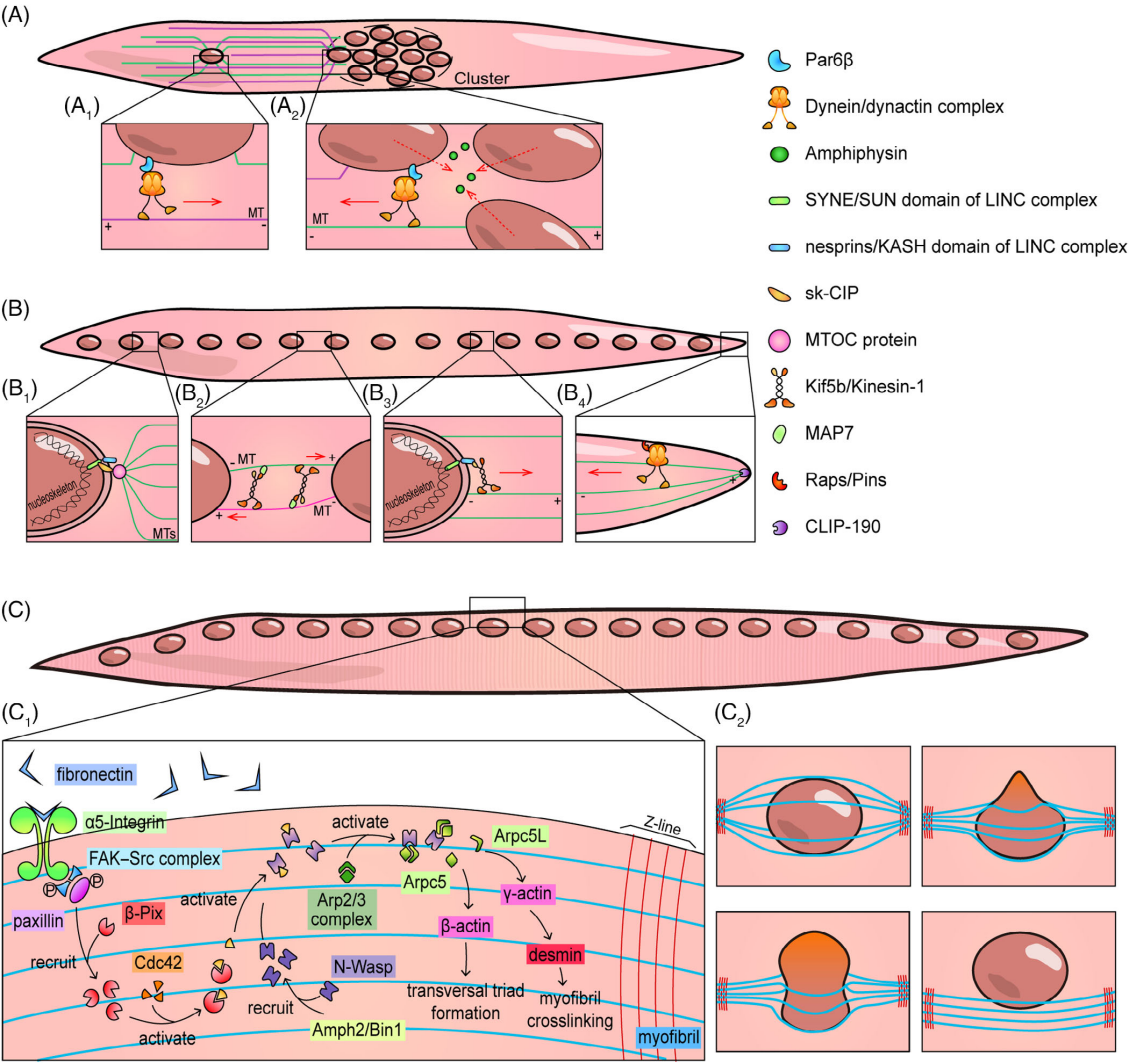
Molecular mechanism of myonuclear migration. Myonuclear migration has three main stages: cluster formation, spreading, and dispersion. (A) Cluster formation is mainly mediated by the dynein/dynactin complex. Dynein/dynactin complex links MTs and myonuclei via Par6β, pulling the newly fused myonuclear towards the cluster. Amphiphysins maintain cluster aggregation. (B) Myonuclei spreading is related to kinesin‐1 and dynein function. Myonuclei emit MTs via the SYNE–sk‐CIP–MTOC protein. Adjacent myonuclei can move on these MTs through the antiparallel MT–Kif5b–MAP7–MT, nuclear–nesprin–KLC–MT, and myotube pole–Raps/Pins–dynein–MT modes. MTs are fixed on the myotube pole using CLIP‐190 in the myotube pole–Raps/Pins–dynein–MT mode. (C) Myonuclei dispersion is based on myofibril crosslinking. Myofibril crosslinking is initiated by fibronectin secreted by myofibroblasts, which interacts with α5‐integrin and phosphorylates FAK–Src complex and paxillin to recruit β‐Pix. Then, Cdc42, N‐Wasp, and Arp2/3 complexes are sequentially activated by the former to promote the function of γ‐actin and desmin, completing myofibril crosslinking. Subsequently, the myonuclei are squeezed to the periphery by myotube contraction. Par6, partitioning defective 6; LINC, linkers of the nucleoskeleton and cytoskeleton; sk‐CIP, skeletal muscle‐cardiac islet‐1 interaction protein; MTOC, microtubule organizing center; MAP7, microtubule‐associated protein 7; Raps, rapsynoid; Pins, partner of inscuteable; CLIP‐190, cytoplasmic linker protein 190; FAK, focal adhesion kinase; β‐Pix, PAK‐interacting exchange factor‐β; Cdc42, cell division cycle 42; N‐Wasp, neural Wiskott‐Aldrich syndrome protein; Amph2, Amphiphysin‐2; Bin1, bridging integrator 1; Arp, actin‐related protein.

Newly added myonuclei, through myoblast–myotube fusion, move to the center of the myotube and form a cluster. The dynein/dynactin complex, Cdc42, Par6, and Par3 were the first molecules to be elucidated in the process of cluster formation.^72^ During the activation of the Pho‐GTPase Cdc42, Par6 interacts with Par3 to recruit the dynein/dynactin complex to the nuclear envelop.^72^ The recruited dynein/dynactin complex binds with Par6β, moves on MTs emitted from the central myotube nuclei and pulls the newly fused nuclei to the cluster^72^ (Figure 4A). Meanwhile, central myotube nuclei can also move on the MTs emitted from the newly fused nuclei via Par6β‐dynein/dynactin complex to shorten the distance by reaction force^72^ (Figure 4A). In research on myonuclei migration in *Drosophila* embryos, the expression of *Amphiphysin* (a centronuclear myopathy‐linked gene) has been proved to tightly aggregate myonuclei to maintain the cluster shape.^69^ Additionally, during the study of the *Drosophila* four‐finger‐shaped LT muscle embryonic formation processes, researchers observed that the aggregated myonuclei were separated into two closely apposed nuclei clusters before extensive myonuclei spread, which is closely related to the role of the LINC complex and Bocksbeutel (*Drosophila* homologs of Emerin).^70^

Myonuclei spread after the end of the fusion process. This is mainly manifested as nuclei spreading along the long axis of the myotube. The spread of myonuclei, found to rely heavily on MTs, has been extensively studied. Nuclei emit MT through the LINC–CIP–MTOC axis, among which skeletal muscle‐specific CIP (sk‐CIP) serves as a connector between SYNE, the SUN domain of the LINC complex, and the MT organizing center (MTOC) protein^73^ (Figure 4B). At present, kinesin‐1 and dynein are believed to be the two protagonists of myonuclei spreading, and there are two types of mechanisms related to kinesin‐1. The first pattern follows the antiparallel MT–Kif5b–MAP7–MT pattern, moving the nuclei away from each other through MT reserve movement^74^, ^75^ (Figure 4B). MT that binds to MAP7 is the “cargo” of kinesin‐1. The second mechanism depends on the binding of the kinesin light chain (KLC) of kinesin‐1 and nesprins^75^, ^76^, ^77^ (Figure 4B). Nesprins are KASH domains of the LINC complex. Researchers have found a conserved tryptophan acidic (LEWD) motif in various nesprins that can bind KLC.^76^ This binding causes the nuclei to become the “cargo” of kinesin‐1 and move away from one another due to the MTs emitted from the adjacent nuclei. In contrast, dynein mediates myonuclei spreading via a completely different mechanism and is mainly described in *Drosophila*. Dynein can be anchored to the cortex at the myotube poles through Raps/Pins, towing the MTs that are emitted by myonuclei and anchored at the myotube poles through CLIP‐190, a *Drosophila melanogaster* homologue of CLIP170^78^ (Figure 4B). The activation of dynein relies on Lis1 and its upstream molecule Nde1, whereas its localization in the myotube cortex and production of tension are regulated by the mammalian JIP3 *Drosophila melanogaster* homologue Syn and the mammalian JIP1/MAPK8IP1 *Drosophila* homologue Aplip1.^79^, ^80^, ^81^ Among them, Aplip1 is believed to regulate both kinesin and dynein depending on its location; Aplip1 near the myotube poles binds with dynein after experiencing high JNK signal transduction, whereas Aplip1 near the cell center activates kinesin after experiencing low JNK signal transduction and interaction with Raps/Pins.^81^ Therefore, the kinesin‐1‐related mechanism encompasses directly affecting the adjacent myonuclei and should be called “proximal pathway,” whereas the dynein‐related mechanism encompasses playing the role of “cortical pathway,” pulling the myonuclei to the ends of myotube.

The nuclei arranged on the long axis of myotubes disperse to the periphery and are located under the sarcolemma after birth during skeletal muscle development in mice and humans.^75^ Researchers have found that myonuclei dispersion is mediated by the centripetal force produced by myofibril crosslinking and contraction^82^ (Figure 4C). Myofibril crosslinking is triggered by the local accumulation of fibronectin secreted by myofibroblasts, which interacts with α5‐integrin and actives focal adhesion kinase (FAK) and Src in the cytoplasm.^83^ Activated FAK–Src complex phosphorylates paxillin, thereby recruiting PAK‐interacting exchange factor‐β (β‐Pix), which is the activator of Cdc42.^84^ Cdc42 then activates N‐Wasp recruited by Amphiphysin‐2 (Amph2)/Bin1 around T‐tubules to promote desmin remodeling under the function of the seven‐subunit actin‐related proteins‐2/3 (Arp2/3) complex containing Arpc5 and Arpc5L, among which Arpc5L mediates the physiological function of γ‐actin and completes myofibril crosslinking.^82^ Desmin is an intermediate filament protein located near the Z‐line of the sarcomere. According to the results of fluorescence staining, desmin is perpendicular to the myofibril and fixes the myofibril on the Z lines.^82^, ^83^ After myofibril crosslinking, myonuclei are squeezed to the periphery by the force generated by the tightening of myofibrils during myotube contraction (Figure 4C). This article describes in detail the force characteristics of myonuclei being squeezed to the periphery, which were not introduced in this review.^82^

Through this process, myonuclei gradually migrate from the initial aggregation in the cell center to the lower part of the sarcolemma, forming a typical nuclear distribution of myofibers. This positioning method protects the myonuclei from damage caused by the traction force generated during myofiber contraction and relaxation. In addition, the perinuclear protective barrier composed of the LINC complex and cytoskeleton also contributes to myonuclei protection to ensure their stability and function.

#### 3.3.2 Coordination between myonuclei after localization

In mature myofibers, the orderly distribution of myonuclei is the basis of normal muscle movement, and the functional area of myonuclear cells is associated with the overall distribution of muscle nuclei. Each myonuclear domain (MND) innervates a certain area of the cytoplasm to control and regulate transcription, translation, and molecular transport processes in this region, which we call the MND. Theoretically, the sum of all MNDs determines the upper limit of myofiber volume under a certain number of myonuclei; however, the number of myonuclei is proportional to the cell surface area rather than the cell volume,^85^ which may be related to signal transduction and material transport on the cell membrane surface under the regulation of myonuclear cells. Based on this, the optimal fiber number hypothesis was proposed: skeletal myofibers tend to reach a larger volume with a certain surface area to reduce metabolic costs, especially oxygen consumption.^86^, ^87^

Under normal circumstances, MND has a certain transcriptional reserve capacity and can adjust its range to maintain the flexibility of regulation and adaption in the case of adaptive muscle growth. However, the function of this regulation is limited to potent muscle hypertrophy after a high‐intensity workload or resistance‐type exercise training. In this case, myofiber growth mainly depends on MuSC fusion to increase the number of myonuclei. Recent studies have indicated that there is no staging effect between MND expansion and myonuclear increase.^88^, ^89^ As the number of myonuclei changes, the size and function of each MND also change. Notably, when more nuclei are added to myofibers, the diversity and quantity of transcription per myonuclear cell are reduced, although the overall output increases. This may indicate that the cost of multinuclear cooperation within a single myofiber is the reduced transcriptional function of each myonucleus.^90^ The difference in the nuclear import rate in different myonuclei reveals the inherent nuclear difference in myofibers^91^; therefore, the role of each nucleus is not exactly the same. For example, during myofiber hypertrophy after progressive weighted‐wheel running, epigenetic differences exist in genes in resident and MuSC‐derived myonuclei, resulting in different contributions to protein production.^92^ The transcriptional profiles of myonuclei have also been confirmed to be diverse and different throughout the lifespan of mice.^93^

## 4 BIOLOGICAL FUNCTION OF SKELETAL MUSCLE

As an important member of the locomotor system, the skeletal muscle is a voluntary muscle that endows limbs and organs with strength and displacement ability via its biological functions. From a macro perspective, skeletal muscle tissue is involved in many biological processes of the body, such as controlling body movement, maintaining body posture, supporting the entry and exit points, protecting bones and organs, and participating in temperature regulation.^7^ In this section, we briefly introduce the biological function of skeletal muscle from three aspects: force generation and movement, metabolic functions, and endocrine functions.

### 4.1 Force generation and movement

#### 4.1.1 Mechanisms of contraction and relaxation

Myofibrils are an important component of mature myofibers, and their basic unit is sarcomere. The sarcomere is the smallest unit of muscle contraction composed of four different myofilament systems: thick filament, thin filament, titin, and nebulin.^16^ Thick filaments are mainly composed of myosin, which is an enzyme with a head that can hydrolyze ATP and provide the energy required for the cross‐bridge cycling.^94^ Thin filaments are polymerized by different actin isoforms, including troponin containing inhibitory subunit TnI, calcium binding subunit TnC and tropomyosin binding unit TnT, and tropomyosin.^94^ Based on the structure of thick and thin filaments, the sliding filament theory was proposed in 1954 to reveal the principle of skeletal muscle contraction.^95^ Titin, also known as connectin, is a giant protein first reported in 1976, whose N2A domain can interact with many molecules to perform different functions, mainly the assembly and fixation of myosin.^96^, ^97^ Shortly after the discovery of titin, nebulin was purified in 1982, and its related molecular interactions have recently been revealed, including serving as a “molecular ruler” for thin filaments through consistent provision of physical length, stabilizing thin filaments through interaction between the SDxxYK motif and three actin subunits, and indirectly regulating myosin as well as muscle contraction by binding to TnT.^98^, ^99^ Further, there is the existence of triads on the Z‐line of skeletal muscle. A Triad is an important structural foundation for excitation–contraction coupling and is composed of a central T‐tubule, terminal cisternae on both sides, and some specialized proteins, such as DHPR, RyR1, calcium buffering proteins, calcium channel regulators, and SERCA.^100^

Based on the above molecules and structures, excitation–contraction coupling and filament sliding together promote muscle contraction as follows^94^, ^101^: (1) the postsynaptic fast sodium channel SCNA4 is activated, generating and transmitting action potential to triads, (2) DHRP is depolarized and induces extracellular Ca^2+^ influx, which triggers a large amount of Ca^2+^ release from terminal cisternae through RyR1, (3) Ca^2+^ binds to TnC, causing a conformational change in troponin, (4) tropomyosin moves away, exposing myosin‐binding sites on actin, (5) the energized head of myosin binds to actin at a 45° angle and undergoes conformational changes to a 90° angle with ADP releases, (6) new ATP binds to the myosin head and causes the dissociation of myosin and actin, (7) the hydrolysis of ATP energizes the myosin head, (8) the sarcomere enters the next cross‐bridge cycling and causes sustained muscle contraction if Ca^2+^ continues to bind to TnC; however, if Ca^2+^ is transported back to the sarcoplasmic reticulum (SR), troponin restores its conformation and blocks tropomyosin, leading to muscle relaxation.

#### 4.1.2 Types of muscle fibers

Myosin heavy chain (MyHC) is an important component of myosin and is closely related to muscle contraction properties. There are four subtypes of MyHC: MyHC I, MyHC IIa, MyHC IIb, and MyHC IIx. Based on these, skeletal muscle fibers are divided into type I (slow oxidative), type IIa (fast‐twitch oxidative glycolytic), type IIb (fast‐twitch glycolytic), and type IIx, which can be identified by MyHC gene PCR detection and immunohistochemical staining including single staining with specific antibodies and simpler multiple immunofluorescence staining.^102^ Type I muscle fibers are rich in mitochondria and active aerobic metabolic enzymes but poor in ATPase; therefore, their contraction is slow and lasting and is mainly responsible for endurance movements. On the contrary, type IIb muscle fibers have high active ATPase and glycolytic related enzymes, and more glycogen but poor aerobic capacity, causing them to produce higher contraction speed and stronger force but less optimized fatigue resistance, and playing a vital role in short and powerful movements. The contraction and metabolic characteristics of type IIa muscle fiber are between type I and type IIb, whereas type IIx is similar to type IIb with lower contraction speed and higher oxidative metabolism. Additionally, muscle fibers undergo phenotypic transformation under the influence of nutritional and non‐nutritional factors to meet the requirements of the external environment, following the pattern type I ↔ type IIa ↔ type IIx ↔ type IIb.^103^

### 4.2 Metabolic functions

Skeletal muscle contraction is a highly ATP‐consuming process, so energy metabolism is essential for the stability and performance of skeletal muscle mechanical function. Under normal circumstances, the ATP reserve in skeletal muscle is relatively low and unable to sustain explosive and prolonged movements. The breakdown of phosphocreatine can rapidly but briefly replenish consumed ATP and maintain muscle concentration when starting physical movements or engaging in short‐term high‐intensity activities.^104^ Subsequently, material metabolic processes, such as glycolytic metabolism/glycolysis, β‐oxidation of fatty acid, and oxidative phosphorylation, are required to continuously generate ATP and maintain muscle contraction. This process is regulated in two stages: overall regulation and fine regulation.^105^ The former is mediated by cellular Ca^2+^, which activates key enzymes, such as phosphorylase kinase, which transforms phosphorylase into the active form and triggers many key steps in metabolism and energy supply using carbohydrates and fatty acids as substrates.^105^, ^106^ Fine regulation refers to adjusting ATP production based on the actual demands, relying on the feedback from ADP, Pi, and other factors related to enzymes. For example, the accumulation of ADP and AMP activates glycogen phosphorylase, which promotes glycogenolysis and ATP supplementation.^105^

#### 4.2.1 Glucose uptake and storage

From the perspective of material metabolism, glucose regulation is the core of energy balance in skeletal muscle and the entire body, and mainly involves glucose uptake, metabolism, and storage. Glucose enters myofiber by facilitating diffusion, and the diffusion rate depends on the number of GLUT4 on the plasma membrane and the concentration gradient of glucose.^107^ In the resting state, GLUT4 is mainly localized in intracellular storage sites rather than sarcolemma and T‐tubules. Muscle contraction and insulin can activate signaling molecules TBC1D1, TBC1D4, and Rac1 via different upstream pathways to stimulate GLUT4 translocation and increase intracellular glucose content for movement consumption or energy storage.^108^ With exercise, skeletal muscle hyperemia and capillary recruitment also promote glucose uptake. The speed and pattern of glucose metabolism in skeletal muscle fibers are not the same. As previously mentioned, from types I to IIb, glucose metabolism gradually changes from oxidative metabolism to glycolysis, resulting in different ATP production rate and muscle contraction speed (I < IIa < IIx < IIb) as well as fatigue resistance capability (I > IIa > IIx > IIb) to meet the demands of different exercises. Additionally, glucose can be stored in skeletal muscle in the form of muscle glycogen, and its storage and depletion are considered to be heterogeneous and fiber‐type specific.^109^ In addition to mobilizing and providing energy in time, glycogen deposited in specific compartments of myofiber also undertakes special regulatory function. For example, the glycogen that is proximate to triad can directly influence excitation–contraction coupling and muscle contraction, and intermyofibrillar glycogen promotes cross‐bridge cycling and SR Ca^2+^ uptake.^109^

#### 4.2.2 Role in thermogenesis

Thermogenesis is a direct reflection of energy metabolism. Skeletal muscle is the main thermogenic organ of the body and has two forms of thermogenesis: shivering thermogenesis and nonshivering thermogenesis (NST).^110^ The former is caused by repetitive involuntary contractions and is activated when suddenly exposed to cold environment. During muscle shivering, heat is derived from ATP hydrolysis, but the long‐term persistence of this process can result in muscle glycogen depletion and muscle fatigue, leading to reduction in heat production efficiency. In cold adaptation, the NST of skeletal muscle and basal adipose tissue gradually occupy a dominant position of thermogenesis. Skeletal muscle‐associated NST relies on SERCA pump‐induced Ca^2+^ entry into SR and accompanying ATP hydrolysis.^111^ Sarcolipin (SLN) can bind to SERCA and block Ca2+ transport to SR without affecting ATP hydrolysis, thereby releasing the energy generated during this process in the form of heat.^112^ This SERCA pump and SLN activity as well as Ca^2+^ cycling do not cause muscle contraction, but are essential for heat production to adapt to long‐term cold exposure. Additionally, skeletal muscle‐associated NST is an important component of diet‐induced thermogenesis. Researches have shown that SLN can promote energy consumption by increasing oxidative metabolism and its overexpression in mice can resist obesity caused by a high‐fat diet.^113^

### 4.3 Endocrine functions

With the discovery of IL‐6 and other factors secreted by skeletal muscle into the blood, skeletal muscle was proved to be a secretory organ with endocrine function. Subsequently, the concept of myokines was proposed, referring to cytokines and other polypeptides synthesized and released by muscle fibers to exert autocrine, paracrine, or endocrine effects.^114^ Currently, more than 650 types of myokines, which play an essential role in regulating muscle function, maintaining metabolic homeostasis, and linking skeletal muscle with other tissues and organs, have been identified.^115^

Some of these myokines regulate the mass and metabolism of skeletal muscle in an autocrine or paracrine manner. Among them, IL‐4, IL‐6, IL‐7, IL‐15, LIF, and musclin have positive effects on muscle hypertrophy, whereas myostatin is the opposite.^116^ In terms of metabolism, IL‐6 increases insulin‐stimulated glucose intake and promotes GLUT4 translocation, and BDNF acts on fatty acid oxidation by activating AMPK.^116^ In addition to the role played by the skeletal muscle, myokines also act on distant organs. In the central nervous system, the improvement of cognitive function by exercise is partially mediated by cathepsin B and irisin, which can cross the blood–brain barrier, stimulate the production of BDNF, and ultimately lead to hippocampal neurogenesis.^117^, ^118^ For adipose tissues, IL‐6 can stimulate visceral fatty lipolysis, and the IL‐6 autoantibody has been implicated in the pathogenesis of type 2 diabetes.^119^ In rodents, IL‐6, meteorin‐like, and irisin have been proven to cause browning of the white adipose tissue.^120^ In the digestive system, myokines act on the gastrointestinal track, islet β cells, and so on to affect liver glucose metabolism and insulin secretion.^121^ Moreover, bone formation is under the regulation of myokines, where IL‐6, decorin, IGF‐1, and FGF‐2 play promoting roles and myostatin plays an inhibitory role.^122^ Additionally, skeletal muscle also interacts with skin, vascular system, immune system, and even cancer through the endocrine effect of myokines.^121^ By and large, the exploration of myokines function has gradually revealed the effect of exercise on various functions of the body and promoted the understanding of relevant diseases.

## 5 ATROPHY, HYPERTROPHY, AND REGENERATION OF SKELETAL MUSCLE

In adulthood, the skeletal muscle exhibits a relatively stable state and functions in an orderly manner under the control of the neural system. However, disturbance of skeletal muscle homeostasis by internal or external stimuli leads to atrophy or hypertrophy, which can occur as an adaptive change under physiological conditions or a damage under pathological conditions. Skeletal muscle can initiate regeneration processes for self‐repair to maintain its size and function in the face of injuries caused by trauma, disease, and infection. In this section, we focus on the response to stimulation of skeletal muscle to discuss characteristics of skeletal muscle in adulthood.

### 5.1 Muscle atrophy and hypertrophy

Muscle plasticity refers to the ability of a muscle to increase, decrease, or maintain a stable muscle mass to better adapt to internal or external environmental changes. Plasticizing is mainly based on the balance between the synthesis and degradation of muscle proteins. Overall, when protein synthesis is less than degradation, skeletal muscles are in an atrophied state; otherwise, they are in a hypertrophic state (Figure 1). The main molecular pathways that control and regulate muscle state include the insulin/IGF1–AKT–mTOR and the TGF‐β/myostatin/BMP pathways that promote protein synthesis and degeneration, respectively.^8^, ^9^ Notably, AKT–mTOR has been shown to be the central pathway affecting muscle proteins, which can be regulated by several upstream factors, such as IGF1, β‐adrenergic, and myostatin/BMP.^123^ Additionally, miR‐106a‐5p can bind to the 3′‐UTR of PIK3R1 to depress the PI3K–AKT pathway, thereby inhibiting MuSC differentiation and promoting muscle atrophy.^124^ However, protein synthesis and degradation are not completely increased or reduced during muscle atrophy and hypertrophy, respectively. For example, amino acids released by the lysosomes and proteasomes after protein breakdown can activate mTOR and promote protein synthesis during muscle atrophy.

Muscle atrophy refers to the loss or thinning of the muscle tissue, mainly caused by a decrease in muscle mechanical load. Besides, inflammation, oxidative stress, and certain proinflammatory cytokines such as TNF‐α are stimulating factors of muscle atrophy and can accelerate the atrophy process.^10^, ^125^ Uncomplicated disuse atrophy is a common adaptive change induced by decreased voluntary physical activity. This type of atrophy is mainly characterized by reduced protein synthesis and no significant increase in protein degradation, with almost no stimulation factors, such as inflammatory factors and hormones, but the relevant mechanisms are still unclear.^126^, ^127^ Some scholars speculate that this anabolic resistance is associated with the reduction of translation efficiency mediated by mTORC1‐independent mechanisms, which still needs further exploration.^126^ Sarcopenia induced by aging, chronic inflammation, and longer‐term inactivity manifests as the coexistence of decreased protein synthesis and increased protein degradation, which is caused by activation of the ubiquitin–proteasome pathway, autophagy–lysosome pathway, caspase system, and calpain system.^125^ In the ubiquitin–proteasome system, researchers have found that the expression of the ubiquitin ligases MuRF1 and MAFbx/Atrogin‐1 is stable and highly upregulated in different muscle atrophy models, and TRAF6 also plays an important role in starvation‐induced muscle atrophy.^128^, ^129^ The transcriptional regulator FoxO3 can activate both the ubiquitin–proteasome and autophagy–lysosome systems, whereas its function can be inhibited by Akt‐mediated phosphorylation.^9^ Additionally, other signaling molecules and pathways, such as FoxOs‐atrogenes, calcineurin pathway, TNFα–IKK–IKB–NF‐κB, and IL‐6–JAK–Stat3, are also involved in the regulation of muscle loss.^8^

Current studies focusing on skeletal muscle hypertrophy are primarily aimed at myogenesis after resistance exercise and muscle repair after atrophy. IGF1 is a crucial growth factor that induces muscle hypertrophy by activating the PI3K–AKT–mTOR and PI3K–AKT–GSK3 pathways. Rapamycin, an mTOR blocker, can inhibit protein synthesis to block hypertrophy.^130^, ^131^ Furthermore, follistatin, androgen, β2‐agonits, and osteocalcin are essential prohypertrophic hormones and growth factors, whereas MEF2, SRF, PGC‐α4, and YAP serve as transcription factors and coactivators that regulate the process of muscle hypertrophy.^123^ In addition to the promotion of protein synthesis and the inhibition of protein degradation, studies have shown that an increased number of myonuclei is essential for long‐term muscle hypertrophy induced by MuSC fusion.^11^

In recent years, it has been confirmed that MuSC‐mediated cell–cell fusion is necessary for the sustainable development of muscle hypertrophy by constructing animal models that destroy MuSC function in vivo.^11^ However, in MuSC‐deficient animal models, the skeletal muscle also shows a certain degree of hypertrophy due to the myomuclei transcriptional reserve capability and the expansion of MNDs.^132^, ^133^ Therefore, MuSCs serve as a reserve force for continuous muscle hypertrophy by increasing myonuclei and may be age related.^134^ Unlike muscle regeneration, there is no muscle damage, and stimulation is mostly induced by resistance exercise. Mechanical tension and load acting on myofiber do not directly induce the fusion process but are essential for driving MuSC proliferation and activation.^135^, ^136^ Mature myofibers secrete NO, IL‐4, and IL‐6 to recruit MuSCs and promote fusion after mechanical stimulation.^46^, ^137^, ^138^, ^139^ In addition to transmitting signals in a paracrine manner, physical cellular interactions between MuSCs and myofibers can promote direct communication through F‐actin‐based tunneling nanotubes.^140^ Surprisingly, macrophages play a role in the activation of MuSCs even in the absence of myofiber damage. RhoA secreted by myofibers can promote the release of the chemokines Ccl3 and Cx3cl1 and recruit macrophages to cope with the short but strong inflammatory response after muscle overload.^141^ Recruited macrophages secrete MMP14 to reshape the extracellular matrix and create conditions for muscle growth during hypertrophy.^142^ The function of the myofiber–macrophage axis in MuSC fusion during muscle hypertrophy deserves further exploration.

### 5.2 Muscle regeneration

Muscle regeneration is the renewal mechanism of multinuclear myofibers following muscle atrophy or damage. Cardiotoxin‐mediated damaged muscle in mice was reconstructed to its original size in approximately 2−3 weeks; therefore, muscle regeneration could be a relatively long process, during which researchers have found that MuSC‐mediated myofiber fusion is essential and irreplaceable.^25^ Myofiber fusion shows two fusion stages during muscle regeneration: the myoblast–myoblast fusion stage and the myoblast–myotube fusion stage, similar to muscle development. However, MuSCs are normally in a resting state after muscle growth arrest; therefore, activators are required to stimulate the proliferation and differentiation of MuSCs to restore their fusion ability. Damaged or dead myofibers secrete various molecules that induce the activation of MuSCs. Tenascin‐C released by necroptotic myofibers activates the EGFR signaling pathway to promote MuSC activation.^143^ Metabolic enzymes, such as GADPH, are damaged myofiber‐derived factors that induce MuSCs to enter and stay in the G1 phase, causing reversible activation.^144^ Furthermore, an inflammatory immune response occurs at the site of muscle injury with the aggregation of many immune cells, among which macrophages play an important role in muscle regeneration.^145^, ^146^, ^147^ In addition to clearing dead myofibers to create space for MuSC proliferation, macrophages can activate MuSCs by secreting macrophage‐derived factors and directly contacting them. TWAKE, a member of the TNF superfamily, is mainly released by macrophages and binds to its receptor Fn14, which is expressed on all progenitor cells of the mesenchymal lineage to promote the proliferation and activation of MuSCs.^148^ The secretory protein Adamts1 targets the Notch1 protein on MuSCs to promote the activation of MuSCs.^149^ The TGF‐β superfamily members GDF‐3 and GDF‐15, upregulated by transcriptional factor RXR/PPARγ, support the timely tissue repair during muscle regeneration.^150^, ^151^ Additionally, direct membrane contact between myogenic cells and macrophages, which persists continuously throughout the myogenic process, has been observed using transmission electron microscopy.^152^ The function of some adhesion molecules changes to meet the demands of regeneration after muscle injury.^153^ Membrane contact allows the direct intercellular transport of soluble molecules and suggests the presence of intermembrane channels. In addition, during the early stage of muscle regeneration, fibroadipogenic progenitors are activated and secrete IL‐6, IL‐33, WISP1, Follistatin, and IL‐10, providing a favorable environment for MuSC proliferation before macrophage function.^154^ Partial reprogramming of muscle tissue induced by systemic and muscle local expression of Yamanaka factors (Oct‐3/4, Sox2, Klf4, and c‐Myc [OSKM]) reshapes the MuSCs niche, promotes the activation of MuSCs, and improves the vitality of regeneration muscles during aging.^155^, ^156^

Notably, there are differences in the characteristics of molecular expression and myonucleus localization between muscle regeneration and muscle hypertrophy.^157^ In overloaded muscle tissue, MuSCs proliferate between myofibers and the basal membrane for cell fusion after activated by stimulation.^158^ Proliferating MuSCs have the characteristics of continuous expression of HEYL and Col5a1, as well as low or no expression of MyoD, which may function as a controller of fusion time by repressing the expression of myomakers.^157^, ^158^ MuSC‐derived nuclei alter the myonuclear methylation profile and preferentially provide ribosomal proteins, indicating that the newly added nuclear and intrinsic nuclei have different transcriptional capabilities.^92^ Furthermore, newly added and MuSC‐derived nuclei do not seem to undergo the process of central gathering, spreading around, and migrating to the periphery, but directly localize in the periphery to function.^159^ But research in recent years has shown that the similar process of myonuclei localization also exists in the middle‐late stage of regeneration, suggesting that there may be no difference between muscle hypertrophy and regeneration at this point.^160^

Researchers have indicated that the skeletal muscle regeneration ability decreases with age owing to the loss of the number and/or function of MuSCs. Reduced Notch signaling and increased Wnt signaling inhibit the regeneration potential of MuSCs.^161^, ^162^ Excess TGF‐β released by aging muscle tissue stimulate the expression of TGF‐β pSmad3 in MuSCs to change MuSCs from a reversible resting state to an aging state.^163^ In addition, the activation of JAK–STAT signaling, overexpression of FGF2, and disinhibition of p16^INK4a^ are all involved in the loss of function of MuSCs.^164^, ^165^, ^166^ Muscle regeneration disorders caused by MuSC dysfunction in the muscle tissue of older individuals may promote the development of sarcopenia to some extent; however, this conclusion needs more experimental data to support. These molecules and signaling pathways are also expected to be targets for restoring muscle regeneration and treating relevant diseases.

## 6 MYOPATHY

**FIGURE 5:**
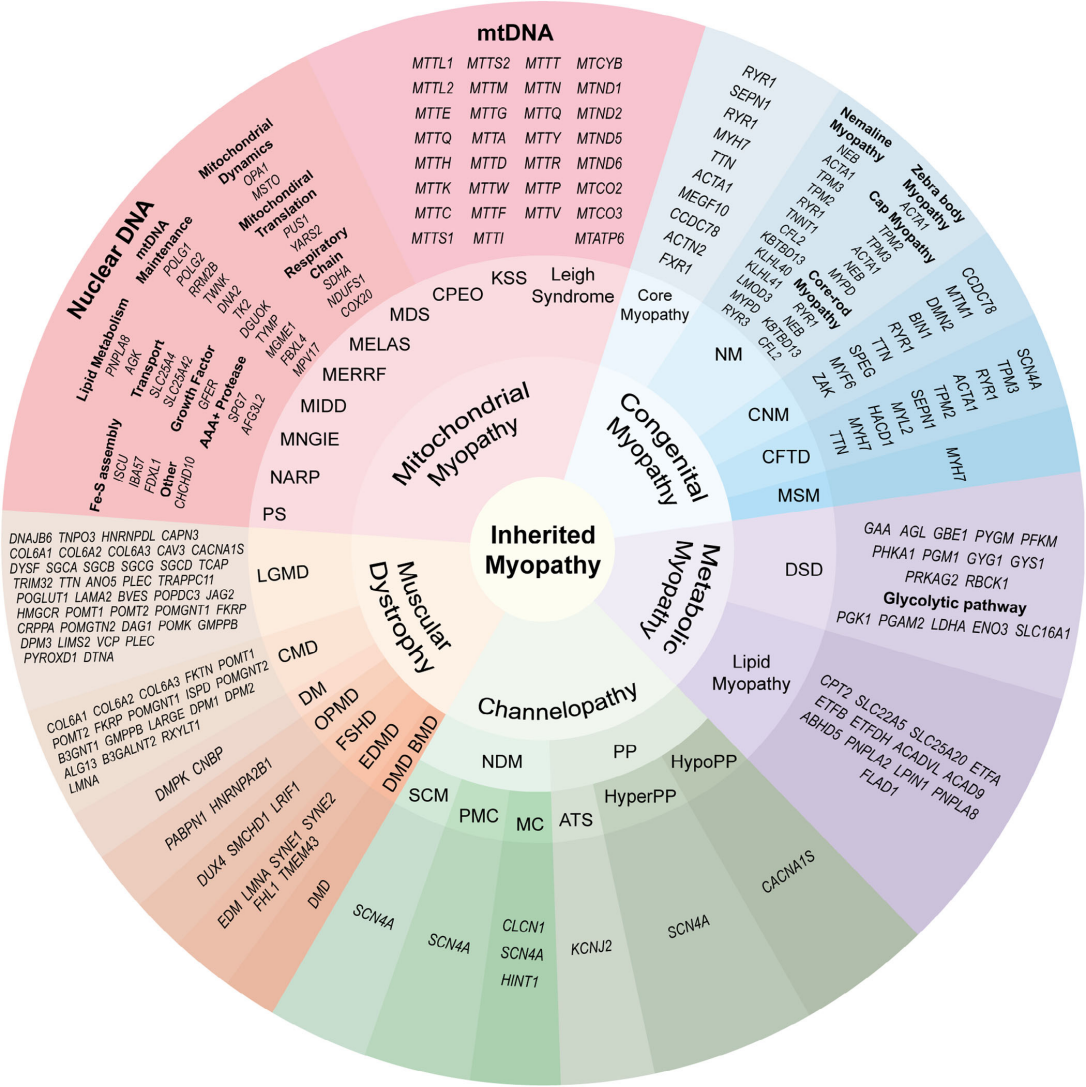
Classification and pathogenic genes of inherited myopathies. Inherited myopathies are divided into five categories: mitochondrial myopathies, congenital myopathies, metabolic myopathies, channelopathies and muscular dystrophies. Mitochindrial myopathies: Kearns–Sayre syndrome (KSS), chronic progressive external ophthalmoplegia (CPEO), leigh syndrome, mitochondrial DNA depletion syndrome (MDS), mitochondrial encephalomyopathy, lactic acidosis and stroke like episodes (MELAS), myoclonus epilepsy with ragged red fibers (MERRF), maternally inherited deafness and diabetes (MIDD), mitochondrial neurogastrointestinal encephalomyopathy (MNGIE), neuropathy, ataxia, and retinitis pigmentosa (NARP), and Pearson syndrome (PS). Congenital myopathies: core myopathy, nemaline myopathy (NM), centronuclear myopathy (CNM), congenital fiber type disproportion (CFTD), and myosin storage myopathy (MSM). Metabolic myopathies: glycogen storage disease (GSD) and lipid myopathy. Channelopathies: nondystrophic myotonias (NDM) including myotonia congenita (MC), paramyotonia congenita (PMC), and sodium channel myotonia (SCM); periodic paralysis (PP) including hypokalemic periodic paralysis (HypoPP), hyperkalemic periodic paralysis (HyperPP), and Andersen–Tawil syndrome (ATS). Muscular dystrophies: Duchenne muscular dystrophy (DMD), Becker muscular dystrophy (BMD), myotonic dystrophy (DM), facioscapulohumeral muscular dystrophy (FSHD), oculopharyngeal muscular dystrophy (OPMD), congenital muscular dystrophy (CMD), Limb‐Girdle muscular dystrophy (LGMD), and Emery–Dreifuss muscular dystrophy (EDMD).

Myopathy is a kind of disease that affects the structure, function, and metabolism of skeletal muscle and can be divided into inherited myopathies and acquired myopathies according to etiology. Inherited myopathies are divided into five categories: mitochondrial myopathies, congenital myopathies, metabolic myopathies, channelopathies, and muscular dystrophies, and more than 200 gene mutations have been identified as the cause of these myopathies^167^, ^168^, ^169^, ^170^, ^171^, ^172^ Acquired myopathies usually have clear predisposing factors, including toxin myopathies caused by drug or toxin poisoning, immune‐mediated or idiopathic inflammatory myopathies, infectious myopathies, endocrine myopathies, electrolyte‐mediated myopathies, and systemic disease‐related myopathies.^173^ Acquired myopathies are not the focus of our discussion. The classification of inherited myopathies and their respective pathogenic genes are listed in detail in Figure 5. In this section, we only summarize myopathies associated with the above molecules and biological processes.

### 6.1 Myopathies related to myomaker and myomerger

The discovery of myomakers and myomergers as well as the deepening of research on fusion processes has gradually expanded our understanding of the pathogenesis of several myopathies. Recent studies have shown that mutations in myomakers and myomergers are associated with several diseases. Carey–Fineman–Ziter syndrome (CFZS) was first reported in 1982.^174^ CFZS is a congenital myopathy with clinical manifestations such as hypotonia, Moebius sequence, Robin sequence, facial anomalies, motor delays, and growth failure.^175^ Recently, researchers found that CFZS is associated with a recessive mutation in the myomaker gene through muscle biopsy and whole‐genome sequencing of patients, which was verified in a zebrafish animal experiment.^176^, ^177^ Mutations in myomergers are also involved in CFZS, and deletion of its extracellular region (MYMX R46* variant) can result in the loss of cell fusion ability and the occurrence of disease in mice.^178^ Additionally, muscle atrophy and abnormal athletic ability caused by spinal muscular atrophy are associated with the decreased expression of myomakers and myomergers, leading to myofiber fusion disorders.^179^ Recently, an interesting argument has been made that the genetic basis of human disease can be revealed through leverage comparisons among species.^180^ Evolutionary homology between myomerger and FAST protein from Reoviridae family may provide ideas for genetic characteristic of some myopathies.^64^, ^181^ Based on these findings, cell–cell fusion deficits can become a new spectrum of congenital myopathy, providing new explanations and therapeutic targets for more diseases.

The discovery of myomakers and myomergers has greatly promoted the study of myofiber fusion and understanding of myopathies. Myomakers and myomergers are only expressed on differentiated MuSCs during skeletal muscle hypertrophy and regeneration (details described below), providing natural targets for disease treatment. Recently, the advent of viral vehicles specifically targeting stimulated or damaged muscle tissues has brought the gospel to gene‐targeted therapy for muscle diseases. Researchers have replaced the fusogens of lentiviruses with myomakers and myomergers and engineered a viral vector that successfully targeted cells expressing myomakers and myomergers, which activated MuSCs and regenerated myofibers in cardiotoxin injury, synergistic ablation, and Duchenne muscular dystrophy models.^182^ Additionally, the viral vector could transduce the dystrophin gene *μDys5* into the genome of myofibers under immune evasion and showed good results after local muscle injection and systemic administration.^182^ Moreover, the expression rate of μDys5 in myofibers was as high as 77−90% in the diaphragm after three systemic doses.^182^ Therefore, it is feasible to solve the targeting problem of viral vectors using skeletal muscle‐specific fusogens and accurately deliver genes or drugs to the corresponding cells. This provides the possibility of permanently modifying MuSCs in skeletal muscle tissue to treat congenital myopathies caused by genetic defects at the genetic level, which is conducive to the precise positioning treatment of myopathies. We look forward to the practical clinical application of targeting the skeletal muscle tissue by replacing the viral fusogen with myomakers and myomergers.

### 6.2 Centronuclear myopathy

Centronuclear myopathy is a rare inherited neuromuscular disease. It is a type of congenital myopathy characterized by a myonuclei located in the center of myofibers upon muscle biopsy. Based on the genetic characteristics and clinical manifestations, centronuclear myopathy is mainly classified into four types: X‐linked recessive centronuclear myopathy (also called myotubular myopathy, XLMTM), autosomal dominant centronuclear myopathy (ADCNM), autosomal recessive centronuclear myopathy (ARCNM), and sporadic centronuclear myopathy.^183^ At present, mutations of seven genes, including *MTM1* encoding myotubularin 1, *DNM2* encoding dynamin 2, *BIN1* encoding amphiphysin 2*, RYR1* encoding the skeletal muscle ryanodine receptor*, TTN* encoding titin, *CCDC78* encoding coiled‐coil domain‐containing protein 78, and *SPEG* encoding striated muscle preferentially expressed protein kinase,^184^, ^185^, ^186^ have been confirmed to be associated with its pathogenesis. *MTM1* mutations are responsible for XLMTM. Dominant mutations of *DNM2* and *BIN1* are related to ADCNM, and recessive mutations of *BIN1*, *RYR1*, and *TTN* are associated with ARCNM. CCDC78 mutations and SPEG mutations are implicated in centronuclear myopathy 4 and centronuclear myopathy 5, respectively. However, approximately 16% of cases have unknown genetic causes and are thus temporarily classified as sporadic centronuclear myopathy.^187^ Here, we discuss XLMTM as it has the highest incidence rate (57% of the total) and exhibits the most serious clinical symptoms.^187^

XLMTM is mainly caused by a recessive mutation in *MTM1* located on Xq28, which is highly conserved from yeasts to humans.^188^ Myotubularin is a phosphosinositide phosphatase that participates in the regulation of intracellular vesicle transport, membrane identity, and protein recruitment by phosphorylating phosphosinositide.^189^, ^190^ According to the Human Gene Mutation Database (databases.lovd.nl/shared/genes/MTM1), as of November 2023, the total number of public *MTM1* variants reported was as high as 596, and the mutations are distributed throughout the coding sequence. XLMTM is one of the most severe groups with high mortality.^191^ The annual incidence rate of XLMTM is about 1 in 50,000 in newborn males^183^; 80% of male patients show severe skeletal muscle weakness, respiratory weakness, and dysphagia at birth, and more than 50% of them will die within 18 months if not treated properly.^192^ However, for male survivors, the progression of XLMTM is relatively slow.^193^ Other symptoms, such as moderate ptosis, ophthalmoplegia, and scoliosis, are also observed in patients.^183^ More than half of the patients eventually die from respiratory failure.^191^ Female XLMTM carriers may also show clinical symptoms with varying severity from severe neonatal forms similar to those in male XLMTM patients to milder adult forms, and the number of symptomatic carriers has been greatly underestimated.^194^ Severe neonatal forms are speculated to be associated with asymmetric X‐chromosome inactivation and X‐chromosome structural changes.^194^ In addition, XLMTM patients carrying *SPEG* mutation have a higher probability of dilated cardiomyopathy due to the interaction between MTM1 and SPEG as well as the wide distribution of SPEG in cardiomyocytes.^186^ At present, the clinical treatment is mainly based on supportive treatment, among which respiratory management is the most common, such as clearing respiratory secretions, providing ventilation support, and preventing respiratory infections.^195^ Other therapies are now under development, several of which have entered the clinical research stage, including tamoxifen drug treatment and adeno‐associated virus (AAV)‐mediated gene replacement therapy.^196^, ^197^, ^198^ It is hoped that these therapies will bring hope to patients.

### 6.3 Muscular dystrophies

Muscular dystrophy is a group of hereditary muscle degenerative disorders characterized by progressive muscle weakness. At present, several genes have been identified to be involved in its pathogenesis, and the proteins they encode are quite extensive, mainly including extracellular matrix and basement membrane proteins, sarcolemma‐associated proteins, enzymes, nuclear membrane proteins, sarcomeric proteins, and endoplasmic reticulum proteins.^171^

Among all types of muscular dystrophy, Duchenne (pseudohypertrophic) muscular dystrophy (DMD) has the highest incidence rate in children, and the prevalence rate in males is about 1 out of 100,000.^199^ DMD is caused by a recessive mutation of the dystrophin gene (*DMD*) located on Xp21.^200^ One third of the cases are caused by sporadic mutations, whereas the remaining results from X‐linked recessive inheritance. Similarly, the pathogenic gene of Becker (Benign Pseudohypertrophic) muscular dystrophy is the allele of *DMD*, and the differences between the two are mainly the later age of onset, slower development, and better prognosis of Becker muscular dystrophy.^201^

Muscular dystrophies resulting from autosomal dominant inheritance include myotonic dystrophy (DM), facioscapulohumeral muscular dystrophy (FSHD), and oculopharyngeal muscular dystrophy (OPMD). The incidence of MD is the highest in adults, and the prevalence rate in the general population is 9.99 per 100,000, with myotonic dystrophy type 1 (DM1) accounting for 92.8% of the cases.^202^ DM1 is caused by amplification of the CTG repeat sequence in the 3′UTR of *DMPK*, which is transcribed into an RNA with ribonuclear foci composed of CUG hairpin structures, resulting in downregulation of the Muscleblind‐like family and abnormal stability of CUGBP Elav‐like family member 1.^203^ FSHD usually occurs from the ages of 10 to 30 years, mainly involving the muscles of the face, neck, and shoulder. FSDH is caused by ectopic expression of the normally silent *DUX4* gene located in the D4Z4 region and is divided into FSDH1 and FSDH2 due to the different genetic causes.^204^ In FSDH1, the expression of *DUX4* is affected by the partial deletion of the D4Z4 repeat sequences, and the severity of the disease is associated with the hypomethylation of 4q35 distal D4Z4.^204^, ^205^ OPMD mainly affects the function of extraocular muscles as well as pharyngeal muscles and slowly progresses to ptosis, dysphagia, and proximal limb weakness.^206^ The disease is caused by the abnormal expansion of GCN repeats (11−18 repeats) in exon 1 of *PABPN1*, a homozygous mutation of which can be inherited by autosomal recessive inheritance.^206^ The longer the GCN repeats, the earlier the onset and the more severe the symptoms.^207^ Recently, another gene was found to be associated with OPMD. The frameshift variants of HNRNPA2B1 can induce early‐onset OPMD.^208^

Many muscular dystrophies can be caused by a variety of different genes and be divided into different subtypes according to the different genetic causes and clinical symptoms. Those with the above characteristics are classified as congenital muscular dystrophy (CMD), Limb‐Girdle muscular dystrophy (LGMD), and EDMD. CMD is characterized by a very early onset of muscle weakness, and the onset time is at birth, specifically one year after birth or even before birth. Presently, 27 pathogenic genes have been identified, and the different gene mutations have corresponding disease phenotypes, such as *LMAM2* mutation causing primary merosin deficiency, *COL6* mutation causing Ullrich CMD, and *ITGA7* mutation causing integrin β‐7‐related CMD.^171^ The main mode of inheritance is autosomal recessive inheritance. LGMD also has several disease phenotypes with different pathogenic genes. According to the latest nomenclature, autosomal dominant subtypes are named LGMDD and numbered, and the recessive subtypes are named LGMDR and numbered.^209^ Currently, more than 37 subtypes have been identified, with over 39 gene abnormalities.^209^, ^210^ EDMD is a rare muscular dystrophy and has attracted attention due to its fatal cardiac complications. Nine genes have been proved to be involved in the pathogenesis of EDMD: *EMD* (EDMD1 subtype), *LMNA* (EDMD2 and EDMD3 subtypes), *SYNE1* (EDMD4 subtype), *SYNE2* (EDMD5 subtype), *FHL1* (EDMD6 subtype), *TMEM43* (EDMD7 subtype), *SUN1*, *SUN2*, and *TTN*.^211^ However, no *EDM* and *LMNA* mutations, which are currently considered the most common, are detected in more than 60% of the patients.^212^ Therefore, there are still pathogenic genes to be discovered.

## 7 CONCLUSIONS AND PERSPECTIVES

In this review, we discussed the key processes of skeletal muscle at two stages of myogenesis, including nuclear‐related behaviors in the embryonic and fetal stages and biological functions as well as poststimulation changes in adulthood. The normal function of myofibers relies on the fine regulation of various myofiber life processes, and the key lies in the management of myonuclei, which are precisely regulated by time and space. The accuracy of time is reflected in the timing of the fusion process. Temporal expression of the corresponding transcription factors controls the appropriate timing for myogenic progenitors to express myomakers and myomergers and initiate membrane fusion. The accuracy of space is reflected in the localization of myonuclei, which affects the balance between the transcriptional inhibition of nuclei and material transport efficiency. The concept of MND can partly explain the distribution of myonuclei and the characteristics of RNA and protein transport. However, the theory does not clarify the relationship and conditions between MND expansion and the increase in new myonuclear cells during physiological and/or pathological hypertrophy.

Under physiological conditions, the changes in myofiber state with exercise and other factors reflect the adaptability of the body, whereas muscle atrophy caused by pathological stimulation have a great impact on motor function. In addition to the above changes, many researchers have suggested that myonuclear reduction may occur during muscle atrophy, based on an increase in myonuclei during hypertrophy, which is called nuclear apoptosis. However, there is currently no direct evidence for this nuclear decrease, and there is no reasonable explanation for how myofibers clear apoptotic nuclei without destroying their normal structure and function. From another perspective, if a myonuclear decrease does not exist, myonuclei accumulation and transcriptional inhibition may be the reason for atrophic muscle loss. Whether the function of atrophic muscles can be palliatively restored by guiding the myonuclear decrease is also a question that needs to be considered.

In general, the interpretation of generalized myogenesis of skeletal muscle is helpful in enhancing the understanding of the complete life activities and functions of skeletal muscles and promotes further exploration of the etiology of several myopathies. In recent years, with the continuous improvement in living standards, muscle augmentation has become a demand for an increasing number of healthy people. Although the pathogenesis of various myopathies has become more detailed and accurate, with the deepening of research on the formation process and functional basis of myofibers, drug target identification and drug development are still urgently needed, especially for the treatment of the inherited myopathies. In addition, targeted muscle tissue‐delivery‐related drugs that promote physiological muscle hypertrophy and enhance muscle function have remarkable research value and application prospects.

## AUTHOR CONTRIBUTIONS

*Conceptualization*: L.‐T. F., H. B., and Z.‐N. C. *Investigation*: L.‐T. F. and H. B. *Writing original*: L.‐T. F. and H. B. *Visualization*: L.‐T. F. and H. B. All authors have read and agreed to the published version of the manuscript.

## CONFLICT OF INTEREST STATEMENT

The authors declare that they have no conflict of interest.

## ETHICS STATEMENT

Not applicable.

## Data Availability

The data included in this study are available upon request from the corresponding authors.
